# Dichloridobis{*N*-[(dimethyl­amino)dimethyl­silyl]-2,6-dimethyl­anilido-κ^2^ *N*,*N*′}zirconium(IV)

**DOI:** 10.1107/S1600536809039804

**Published:** 2009-10-07

**Authors:** Juan Chen

**Affiliations:** a Department of Chemistry, Taiyuan Teachers College, Taiyuan 030031, People’s Republic of China

## Abstract

The monomeric title zirconium(IV) compound, [Zr(C_12_H_21_N_2_Si)_2_Cl_2_], was prepared by the metathetical reaction of [LiN(SiMe_2_NMe_2_)(2,6-Me_2_C_6_H_3_)]_2_ with zirconium tetra­chloride. The Zr^IV^ atom is *N*,*N*′-chelated by the *N*-silylated anilido ligand. Along with two Cl atoms, the six-coordinated Zr^IV^ atom demonstrates a highly distorted octa­hedral geometry. The two ligands around the Zr^IV^ atom are arranged *cis* to each other and obey the *C*_2_ symmetry operation. That means the asymmetric unit consists of only half of the mol­ecular compound and the complete mol­ecule is generated by a twofold axis. The two ends of the N—Si—N chelating unit exhibit different affinities for the metal center. The Zr—N_amino_ bond is longer than the Zr—N_anilido_ bond.

## Related literature

For the catalytic applications of related *N*-silylated analido group 4 metal compounds towards olefin polymerization, see: Gibson *et al.* (1998 ▶); Hill & Hitchcock (2002 ▶). For related organometallic compounds supported with analogous analido ligands, see: Schumann *et al.* (2000 ▶); Chen *et al.* (2007 ▶); Ferreira *et al.* (2007 ▶); Chen (2008 ▶).
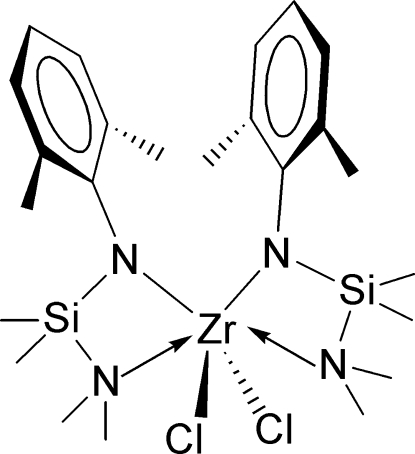


## Experimental

### 

#### Crystal data

[Zr(C_12_H_21_N_2_Si)_2_Cl_2_]
*M* *_r_* = 604.92
Monoclinic, 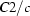

*a* = 16.179 (2) Å
*b* = 10.257 (2) Å
*c* = 18.779 (4) Å
β = 112.234 (4)°
*V* = 2884.6 (9) Å^3^
*Z* = 4
Mo *K*α radiation
μ = 0.67 mm^−1^
*T* = 203 K
0.20 × 0.20 × 0.20 mm

#### Data collection

Bruker SMART area-detector diffractometer
Absorption correction: multi-scan (*SADABS*; Sheldrick, 1996 ▶) *T*_min_ = 0.757, *T*_max_ = 0.878
6014 measured reflections
2548 independent reflections
2448 reflections with *I* > 2σ(*I*)
*R*_int_ = 0.027

#### Refinement

*R*[*F* ^2^ > 2σ(*F* ^2^)] = 0.048
*wR*(*F* ^2^) = 0.120
*S* = 1.20
2548 reflections
151 parameters
H-atom parameters constrained
Δρ_max_ = 0.52 e Å^−3^
Δρ_min_ = −0.53 e Å^−3^

### 

Data collection: *SMART* (Bruker, 2000 ▶); cell refinement: *SAINT* (Bruker, 2000 ▶); data reduction: *SAINT*; program(s) used to solve structure: *SHELXS97* (Sheldrick, 2008 ▶); program(s) used to refine structure: *SHELXL97* (Sheldrick, 2008 ▶); molecular graphics: *SHELXTL/PC* (Sheldrick, 2008 ▶); software used to prepare material for publication: *SHELXL97*.

## Supplementary Material

Crystal structure: contains datablocks I, global. DOI: 10.1107/S1600536809039804/vm2005sup1.cif

Structure factors: contains datablocks I. DOI: 10.1107/S1600536809039804/vm2005Isup2.hkl

Additional supplementary materials:  crystallographic information; 3D view; checkCIF report

## Figures and Tables

**Table 1:** Selected bond lengths (Å)

Zr1—N1	2.119 (3)
Zr1—N2	2.439 (3)
Zr1—Cl1	2.4676 (11)

**Fig. 1.**
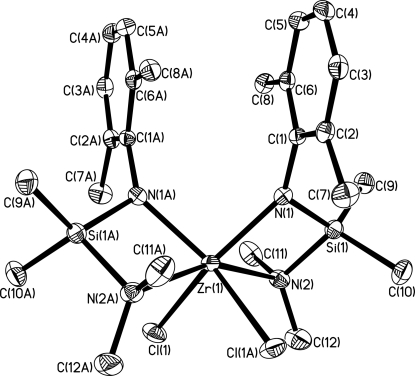
The molecular structure, showing the atom-numbering scheme. Displacement ellipsoids are drawn at the 30% probability level. Hydrogens omitted for clarity. Symmetry codes: (i) -x, y, -z + 1/2.

## supplementary crystallographic information

### Comment

Group 4 metal amides supported with the *N*-silylated anilido ligands were active catalysts for olefin polymerization (Gibson *et al.*, 1998; Hill & Hitchcock, 2002). The *N*-silylated anilido ligand in the title compound has a pendant amino group. It results in an N—Si—N chelating moiety, which is presumed to be a "quasi" conjugated unit owing to *d*–π interaction between Si and N atoms. The zinc compound coordinated with the analogous ligand has been reported by Schumann *et al.* (2000). The title compound is monomeric and contains two *N*-silylated anilido ligands, which give the N—Zr—N bite angle of 67.63° and are arranged *cis* to each other and obey the *C_2_* symmetrical operation. That means the asymmetric unit consists of only half of the molecular compound and the complete molecule is generated by a twofold axis. The Zr^IV^ atom is situated in the plane defined by atoms N1, N2A and Cl1, and the symmetrical counterparts. Both planes are perpendicular to each other resulting in a highly distorted octahedral geometry. Two ends of the N—Si—N chelating unit exhibit different affinity to the metal center. The Zr—N_anilido_ bond is 2.119 Å and the Zr—N_amino_ bond is 2.439 Å, suggesting the former is much tighter than the latter.

### Experimental

ZrCl_4_ (0.47 g, 2.03 mmol) was added into the solution of [LiN(SiMe_2_NMe_2_)(2,6-Me_2_C_6_H_3_)]_2_ (0.92 g, 2.03 mmol) in Et_2_O (30 ml) at 273 K. The reaction mixture was warmed to room temperature and kept stirring for 12 h. It was dried in vacuum to remove all volatiles and the residue was extracted with CH_2_Cl_2_ (30 ml). Concentration of the filtrate under reduced pressure gave the title compound as colorless crystals (yield 0.92 g, 75%).

### Refinement

The methyl H atoms were constrained to an ideal geometry, with C—H distances of 0.97 Å and *U*_iso_(H) = 1.5*U*_eq_(C), but each group was allowed to rotate freely about its C–C, C–N and C–Si bonds. The other H atoms were placed in geometrically idealized positions and constrained to ride on their parent atoms, with C—H distances in the range 0.94Å and *U*_iso_(H) = 1.2*U*_eq_(C).

### Crystal data

**Table:**

[Zr(C_12_H_21_N_2_Si)_2_Cl_2_]	*F*(000) = 1264
*M**_r_* = 604.92	*D*_x_ = 1.393 Mg m^−^^3^
Monoclinic, *C*2/*c*	Mo *K*α radiation, λ = 0.71073 Å
Hall symbol: -C 2yc	Cell parameters from 3803 reflections
*a* = 16.179 (2) Å	θ = 2.3–27.1°
*b* = 10.257 (2) Å	µ = 0.67 mm^−^^1^
*c* = 18.779 (4) Å	*T* = 203 K
β = 112.234 (4)°	Block, colorless
*V* = 2884.6 (9) Å^3^	0.20 × 0.20 × 0.20 mm
*Z* = 4	

### Data collection

**Table:**

Bruker SMART area-detector diffractometer	2548 independent reflections
Radiation source: fine-focus sealed tube	2448 reflections with *I* > 2σ(*I*)
graphite	*R*_int_ = 0.027
φ and ω scans	θ_max_ = 25.0°, θ_min_ = 2.3°
Absorption correction: multi-scan (*SADABS*; Sheldrick, 1996)	*h* = −19→19
*T*_min_ = 0.757, *T*_max_ = 0.878	*k* = −12→12
6014 measured reflections	*l* = −18→22

### Refinement

**Table:**

Refinement on *F*^2^	Primary atom site location: structure-invariant direct methods
Least-squares matrix: full	Secondary atom site location: difference Fourier map
*R*[*F*^2^ > 2σ(*F*^2^)] = 0.048	Hydrogen site location: inferred from neighbouring sites
*wR*(*F*^2^) = 0.120	H-atom parameters constrained
*S* = 1.20	*w* = 1/[σ^2^(*F*_o_^2^) + (0.0465*P*)^2^ + 9.9576*P*] where *P* = (*F*_o_^2^ + 2*F*_c_^2^)/3
2548 reflections	(Δ/σ)_max_ < 0.001
151 parameters	Δρ_max_ = 0.52 e Å^−^^3^
0 restraints	Δρ_min_ = −0.53 e Å^−^^3^

### Special details

**Table:**

Geometry. All e.s.d.'s (except the e.s.d. in the dihedral angle between two l.s. planes) are estimated using the full covariance matrix. The cell e.s.d.'s are taken into account individually in the estimation of e.s.d.'s in distances, angles and torsion angles; correlations between e.s.d.'s in cell parameters are only used when they are defined by crystal symmetry. An approximate (isotropic) treatment of cell e.s.d.'s is used for estimating e.s.d.'s involving l.s. planes.
Refinement. Refinement of *F*^2^ against ALL reflections. The weighted *R*-factor *wR* and goodness of fit *S* are based on *F*^2^, conventional *R*-factors *R* are based on *F*, with *F* set to zero for negative *F*^2^. The threshold expression of *F*^2^ > σ(*F*^2^) is used only for calculating *R*-factors(gt) *etc*. and is not relevant to the choice of reflections for refinement. *R*-factors based on *F*^2^ are statistically about twice as large as those based on *F*, and *R*- factors based on ALL data will be even larger.

### Fractional atomic coordinates and isotropic or equivalent isotropic displacement parameters (Å^2^)

**Table:**

	*x*	*y*	*z*	*U*_iso_*/*U*_eq_	
Zr1	0.0000	0.26497 (5)	0.2500	0.02462 (18)	
Si1	−0.03902 (7)	0.19837 (11)	0.39184 (6)	0.0297 (3)	
Cl1	−0.10371 (7)	0.42525 (10)	0.16362 (7)	0.0427 (3)	
N1	0.01144 (19)	0.1249 (3)	0.33620 (17)	0.0249 (7)	
N2	−0.0992 (2)	0.3112 (3)	0.3175 (2)	0.0337 (8)	
C1	0.0574 (2)	0.0027 (3)	0.3583 (2)	0.0236 (8)	
C2	0.1508 (2)	−0.0007 (4)	0.3993 (2)	0.0278 (8)	
C3	0.1939 (3)	−0.1203 (4)	0.4175 (2)	0.0325 (9)	
H3A	0.2562	−0.1222	0.4439	0.039*	
C4	0.1478 (3)	−0.2359 (4)	0.3977 (2)	0.0345 (9)	
H4A	0.1782	−0.3159	0.4099	0.041*	
C5	0.0562 (3)	−0.2329 (4)	0.3598 (2)	0.0330 (9)	
H5A	0.0245	−0.3118	0.3468	0.040*	
C6	0.0100 (2)	−0.1158 (4)	0.3404 (2)	0.0265 (8)	
C7	0.2058 (3)	0.1209 (4)	0.4260 (3)	0.0416 (11)	
H7A	0.2680	0.0974	0.4527	0.062*	
H7B	0.1851	0.1688	0.4607	0.062*	
H7C	0.1998	0.1750	0.3820	0.062*	
C8	−0.0900 (2)	−0.1221 (4)	0.3002 (2)	0.0314 (9)	
H8A	−0.1059	−0.1936	0.2636	0.047*	
H8B	−0.1118	−0.0409	0.2731	0.047*	
H8C	−0.1169	−0.1360	0.3378	0.047*	
C9	−0.1136 (3)	0.0973 (4)	0.4237 (3)	0.0404 (10)	
H9A	−0.0987	0.1103	0.4783	0.061*	
H9B	−0.1059	0.0061	0.4140	0.061*	
H9C	−0.1752	0.1225	0.3955	0.061*	
C10	0.0358 (3)	0.2854 (5)	0.4793 (3)	0.0458 (11)	
H10A	0.0207	0.2609	0.5229	0.069*	
H10B	0.0284	0.3787	0.4711	0.069*	
H10C	0.0974	0.2618	0.4897	0.069*	
C11	−0.1893 (3)	0.2614 (5)	0.2688 (3)	0.0478 (12)	
H11A	−0.2305	0.2784	0.2941	0.072*	
H11D	−0.1860	0.1683	0.2613	0.072*	
H11C	−0.2100	0.3051	0.2194	0.072*	
C12	−0.1088 (4)	0.4470 (5)	0.3400 (3)	0.0536 (13)	
H12B	−0.1503	0.4492	0.3661	0.080*	
H12C	−0.1312	0.5013	0.2944	0.080*	
H12A	−0.0511	0.4794	0.3742	0.080*	

### Atomic displacement parameters (Å^2^)

**Table:**

	*U*^11^	*U*^22^	*U*^33^	*U*^12^	*U*^13^	*U*^23^
Zr1	0.0262 (3)	0.0198 (3)	0.0309 (3)	0.000	0.0142 (2)	0.000
Si1	0.0310 (6)	0.0305 (6)	0.0303 (6)	0.0019 (4)	0.0148 (5)	−0.0011 (4)
Cl1	0.0491 (6)	0.0310 (6)	0.0538 (7)	0.0120 (5)	0.0259 (6)	0.0139 (5)
N1	0.0206 (15)	0.0262 (17)	0.0273 (17)	0.0006 (12)	0.0086 (13)	−0.0026 (13)
N2	0.0347 (19)	0.0300 (19)	0.041 (2)	0.0072 (15)	0.0194 (16)	0.0046 (15)
C1	0.0258 (18)	0.0221 (19)	0.0225 (18)	0.0028 (15)	0.0086 (15)	−0.0003 (15)
C2	0.0254 (19)	0.033 (2)	0.025 (2)	−0.0016 (16)	0.0107 (16)	−0.0034 (16)
C3	0.028 (2)	0.043 (2)	0.026 (2)	0.0095 (18)	0.0101 (17)	0.0027 (18)
C4	0.040 (2)	0.032 (2)	0.033 (2)	0.0104 (18)	0.0139 (19)	0.0052 (18)
C5	0.041 (2)	0.026 (2)	0.033 (2)	−0.0030 (18)	0.0150 (19)	0.0014 (17)
C6	0.0272 (19)	0.032 (2)	0.0221 (19)	−0.0027 (16)	0.0113 (16)	−0.0016 (16)
C7	0.026 (2)	0.043 (3)	0.047 (3)	−0.0042 (19)	0.0039 (19)	−0.006 (2)
C8	0.028 (2)	0.035 (2)	0.032 (2)	−0.0067 (17)	0.0127 (18)	−0.0042 (17)
C9	0.047 (3)	0.042 (3)	0.041 (3)	0.002 (2)	0.027 (2)	0.002 (2)
C10	0.053 (3)	0.050 (3)	0.034 (2)	−0.005 (2)	0.017 (2)	−0.012 (2)
C11	0.031 (2)	0.060 (3)	0.052 (3)	0.009 (2)	0.016 (2)	0.019 (2)
C12	0.072 (3)	0.039 (3)	0.073 (4)	0.017 (2)	0.053 (3)	0.006 (2)

### Geometric parameters (Å, °)

**Table:**

Zr1—N1^i^	2.119 (3)	C5—C6	1.389 (6)
Zr1—N1	2.119 (3)	C5—H5A	0.9400
Zr1—N2^i^	2.439 (3)	C6—C8	1.506 (5)
Zr1—N2	2.439 (3)	C7—H7A	0.9700
Zr1—Cl1^i^	2.4676 (11)	C7—H7B	0.9700
Zr1—Cl1	2.4676 (11)	C7—H7C	0.9700
Zr1—Si1^i^	3.0385 (12)	C8—H8A	0.9700
Si1—N1	1.724 (3)	C8—H8B	0.9700
Si1—N2	1.791 (4)	C8—H8C	0.9700
Si1—C9	1.854 (4)	C9—H9A	0.9700
Si1—C10	1.859 (5)	C9—H9B	0.9700
N1—C1	1.436 (5)	C9—H9C	0.9700
N2—C12	1.480 (6)	C10—H10A	0.9700
N2—C11	1.487 (6)	C10—H10B	0.9700
C1—C6	1.408 (5)	C10—H10C	0.9700
C1—C2	1.413 (5)	C11—H11A	0.9700
C2—C3	1.388 (6)	C11—H11D	0.9700
C2—C7	1.504 (6)	C11—H11C	0.9700
C3—C4	1.376 (6)	C12—H12B	0.9700
C3—H3A	0.9400	C12—H12C	0.9700
C4—C5	1.379 (6)	C12—H12A	0.9700
C4—H4A	0.9400		
			
N1^i^—Zr1—N1	94.65 (16)	C3—C4—C5	119.2 (4)
N1^i^—Zr1—N2^i^	67.63 (11)	C3—C4—H4A	120.4
N1—Zr1—N2^i^	130.12 (12)	C5—C4—H4A	120.4
N1^i^—Zr1—N2	130.12 (12)	C4—C5—C6	121.4 (4)
N1—Zr1—N2	67.63 (11)	C4—C5—H5A	119.3
N2^i^—Zr1—N2	157.56 (16)	C6—C5—H5A	119.3
N1^i^—Zr1—Cl1^i^	142.64 (8)	C5—C6—C1	119.5 (3)
N1—Zr1—Cl1^i^	96.22 (9)	C5—C6—C8	117.7 (4)
N2^i^—Zr1—Cl1^i^	78.14 (8)	C1—C6—C8	122.8 (3)
N2—Zr1—Cl1^i^	86.92 (9)	C2—C7—H7A	109.5
N1^i^—Zr1—Cl1	96.22 (9)	C2—C7—H7B	109.5
N1—Zr1—Cl1	142.64 (8)	H7A—C7—H7B	109.5
N2^i^—Zr1—Cl1	86.92 (9)	C2—C7—H7C	109.5
N2—Zr1—Cl1	78.14 (8)	H7A—C7—H7C	109.5
Cl1^i^—Zr1—Cl1	96.45 (6)	H7B—C7—H7C	109.5
N1^i^—Zr1—Si1^i^	33.40 (8)	C6—C8—H8A	109.5
N1—Zr1—Si1^i^	122.01 (9)	C6—C8—H8B	109.5
N2^i^—Zr1—Si1^i^	36.12 (8)	H8A—C8—H8B	109.5
N2—Zr1—Si1^i^	153.54 (9)	C6—C8—H8C	109.5
Cl1^i^—Zr1—Si1^i^	114.26 (3)	H8A—C8—H8C	109.5
Cl1—Zr1—Si1^i^	83.61 (4)	H8B—C8—H8C	109.5
N1—Si1—N2	93.02 (16)	Si1—C9—H9A	109.5
N1—Si1—C9	117.81 (19)	Si1—C9—H9B	109.5
N2—Si1—C9	112.64 (19)	H9A—C9—H9B	109.5
N1—Si1—C10	116.49 (19)	Si1—C9—H9C	109.5
N2—Si1—C10	111.0 (2)	H9A—C9—H9C	109.5
C9—Si1—C10	105.6 (2)	H9B—C9—H9C	109.5
C1—N1—Si1	121.2 (2)	Si1—C10—H10A	109.5
C1—N1—Zr1	134.3 (2)	Si1—C10—H10B	109.5
Si1—N1—Zr1	104.01 (15)	H10A—C10—H10B	109.5
C12—N2—C11	108.4 (4)	Si1—C10—H10C	109.5
C12—N2—Si1	118.1 (3)	H10A—C10—H10C	109.5
C11—N2—Si1	112.0 (3)	H10B—C10—H10C	109.5
C12—N2—Zr1	119.4 (3)	N2—C11—H11A	109.5
C11—N2—Zr1	107.3 (3)	N2—C11—H11D	109.5
Si1—N2—Zr1	90.49 (13)	H11A—C11—H11D	109.5
C6—C1—C2	118.9 (3)	N2—C11—H11C	109.5
C6—C1—N1	120.6 (3)	H11A—C11—H11C	109.5
C2—C1—N1	120.6 (3)	H11D—C11—H11C	109.5
C3—C2—C1	119.4 (4)	N2—C12—H12B	109.5
C3—C2—C7	118.1 (3)	N2—C12—H12C	109.5
C1—C2—C7	122.5 (3)	H12B—C12—H12C	109.5
C4—C3—C2	121.6 (4)	N2—C12—H12A	109.5
C4—C3—H3A	119.2	H12B—C12—H12A	109.5
C2—C3—H3A	119.2	H12C—C12—H12A	109.5

Symmetry codes: (i) −*x*, *y*, −*z*+1/2.
